# Chemical Composition and In Vitro Antioxidant Activity of *Salvia aratocensis* (Lamiaceae) Essential Oils and Extracts

**DOI:** 10.3390/molecules28104062

**Published:** 2023-05-12

**Authors:** Juan C. Henríquez, Laura V. Duarte, Lady J. Sierra, José L. Fernández-Alonso, Jairo R. Martínez, Elena E. Stashenko

**Affiliations:** 1 Centro de Cromatografía y Espectrometría de Masas, CROM-MASS, Grupo de Investigación en Biomoléculas CIBIMOL, Universidad Industrial de Santander, Bucaramanga 680002, Colombia; 2 Real Jardín Botánico—CSIC, Claudio Moyano 1, 28014 Madrid, Spain

**Keywords:** *Salvia aratocensis*, essential oil, flavonoids, antioxidant activity, Colombia

## Abstract

*Salvia aratocensis* (Lamiaceae) is an endemic shrub from the Chicamocha River Canyon in Santander (Colombia). Its essential oil (EO) was distilled from the aerial parts of the plant via steam distillation and microwave-assisted hydrodistillation and analyzed using GC/MS and GC/FID. Hydroethanolic extracts were isolated from dry plants before distillation and from the residual plant material after distillation. The extracts were characterized via UHPLC-ESI^(+/−)^-Orbitrap-HRMS. The *S. aratocensis* essential oil was rich in oxygenated sesquiterpenes (60–69%) and presented τ-cadinol (44–48%) and 1,10-di-*epi*-cubenol (21–24%) as its major components. The in vitro antioxidant activity of the EOs, measured via an ABTS^+•^ assay, was 32–49 μmol Trolox^®^ g^−1^ and that measured using the ORAC assay was 1520–1610 μmol Trolox^®^ g^−1^. Ursolic acid (28.9–39.8 mg g^−1^) and luteolin-7-*O*-glucuronide (1.16–25.3 mg g^−1^) were the major *S. aratocensis* extract constituents. The antioxidant activity of the *S. aratocensis* extract, obtained from undistilled plant material, was higher (82 ± 4 μmol Trolox^®^ g^−1^, ABTS^+•^; 1300 ± 14 μmol Trolox^®^ g^−1^, ORAC) than that of the extracts obtained from the residual plant material (51–73 μmol Trolox^®^ g^−1^, ABTS^+•^; 752–1205 μmol Trolox^®^ g^−1^, ORAC). *S. aratocensis* EO and extract had higher ORAC antioxidant capacity than the reference substances butyl hydroxy toluene (98 μmol Trolox^®^ g^−1^) and α-tocopherol (450 μmol Trolox^®^ g^−1^). *S*. *aratocensis* EOs and extracts have the potential to be used as natural antioxidants for cosmetics and pharmaceutical products.

## 1. Introduction

**Figure 1:**
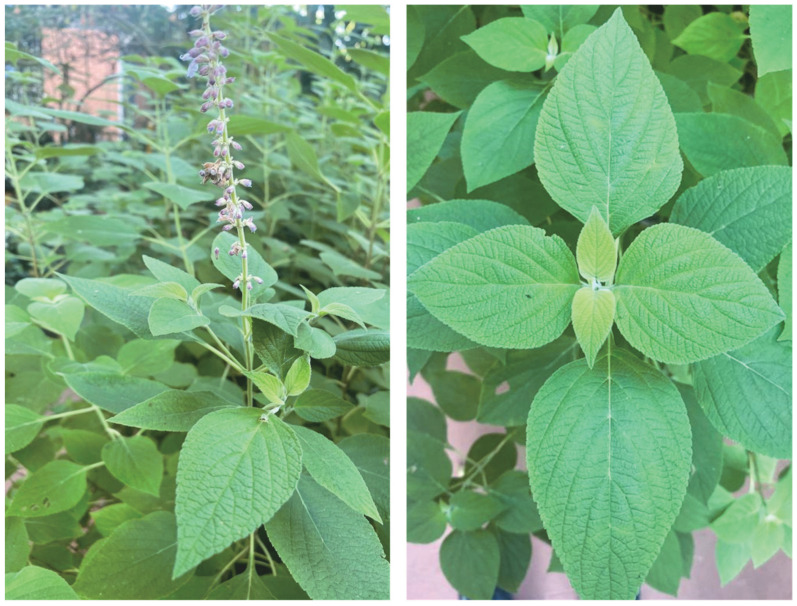
*S. aratocensis* (voucher number COL517740) plants cultivated at the Pilot Agroindustrial Center, CENIVAM-UIS, Bucaramanga, Colombia.

The genus *Salvia* L. belongs to the Lamiaceae family and includes approximately 1000 species distributed throughout the world [1]. *Salvia* plants, in the form of infusions and decoctions, have been traditionally used to treat colds, pain, infections, bronchitis, and insomnia [2]. In the *Vademecum Colombiano de planta medicinales*, only two *Salvia* species have been documented, i.e., *S*. *paliifolia* Kunth, which is a native plant, and *S*. *officinalis* L., which was introduced [3]. In Colombia, 20 sections and more than 75 taxa of *Salvia* have been described, which makes the genus *Salvia* the most diverse of the Lamiaceae family in Colombia [4,5]. *S. aratocensis* (J.R.I. Wood & Harely) Fern. Alonso, a species assigned to the Section *Angulatae* Epling (Figure 1), is a bushy and resistant aromatic plant that grows up to two meters in height and is found mainly between 900 and 2500 MAMSL on the southern slope of the Chicamocha River Canyon in Boyacá and Santander (Colombia) [4,5]. *S. aratocensis* has an essential oil (EO) rich in the oxygenated sesquiterpene τ-cadinol, which possesses antibacterial activity against *Mycobacterium tuberculosis* (MIC = 125 μg mL^−1^) [6].

During the distillation of aromatic plants, two byproducts are generated, i.e., hydrolate and residual biomass; the latter can represent more than 98% of the weight of all the plant material used. The residual biomass contains compounds of biological interest such as flavonoids [7], which are plant secondary metabolites classified as phenolic compounds. Flavonoids participate in the processes of pigmentation, immunization, protection against UV radiation, and nitrogen fixation, among others [8].

The flavones luteolin, cirsimaritin, and eupatilin have been found in extracts isolated from *Salvia* spp. [9,10,11]. The biological activities of flavones are as follows: neuroprotective, anti-inflammatory, antiulcer, antimicrobial, antimalarial, antidiabetic, anticancer, and antioxidant activity, among others [12]. Antioxidants such as flavones are used to prevent cancer, cardiovascular disease, and neurodegenerative disorders because they contribute to the balance between antioxidant defense and the generation of radicals through genetic and environmental means [12]. In a previous study, the antioxidant activity of *S. aratocensis* extracts, measured using the DPPH^•^ assay, was ten times lower than that of vitamin E [13]; however, to date, no reports on the substances present in the extracts have been published.

The main objective of this research was to determine the effect of the distillation processes of *S. aratocensis* via steam distillation (SD) and microwave-assisted hydrodistillation (MWHD) on the EO and extract chemical compositions and on the antioxidant activity of the extracts obtained from plant material before and after distillation.

## 2. Results

### 2.1. Essential Oil and Extract Yields

The yields of essential oils distilled from *S. aratocensis* via SD and MWHD and those of the hydroalcoholic extracts obtained from dry plant material before and after distillation are shown in Table 1.

The EO yields from *S*. *aratocensis* distilled via SD (0.07%) and MWHD (0.08%) were lower than those reported by Bueno et al. [6] (0.5%). The yields of the *S*. *aratocensis* extracts obtained from the dry plants before distillation and those from the dry plant material after SD or MWHD were 19%, 4.6%, and 4.5%, respectively. No previous reports were found on the *S*. *aratocensis* extraction yields either from dry plants before distillation or from residual biomass after distillation.

### 2.2. Essential Oil Chemical Characterization

**Figure 2:**
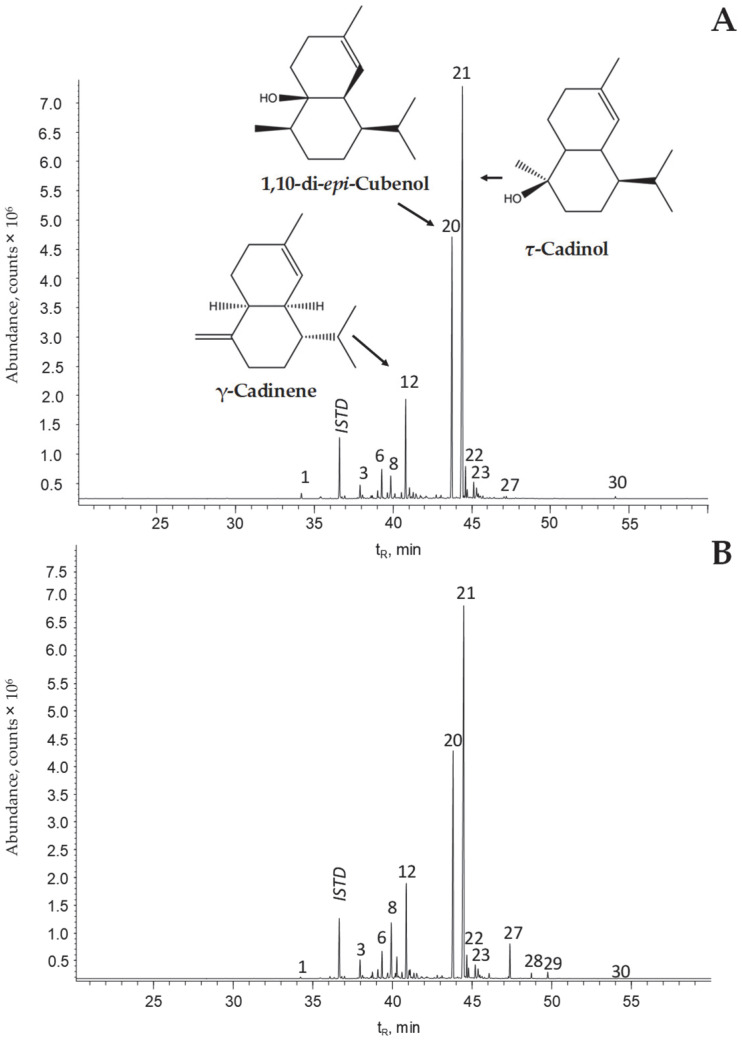
GC/FID-based chromatographic profiles of the EOs distilled from *S. aratocensis* plants via (**A**) hydrodistillation assisted by microwave radiation and (**B**) steam distillation. DB-5 Column (60 m). Split 1:30. ISTD: *n*-tetradecane (0.5 g/L). See the identification of the chromatographic peaks in Table 2.

*S*. *aratocensis* EO analysis identified 28 compounds (Table 2), including 14 sesquiterpene hydrocarbons, 10 oxygenated sesquiterpenes, 2 benzoic acid derivatives, 1 oxygenated monoterpene, and 1 diterpene. Oxygenated sesquiterpenes (60–69%) were the most abundant compounds, followed by sesquiterpene hydrocarbons (24–31%). Figure 2 shows the GC/FID chromatographic profiles of the *S. aratocensis* EOs obtained via SD and MWHD.

**Figure 3:**
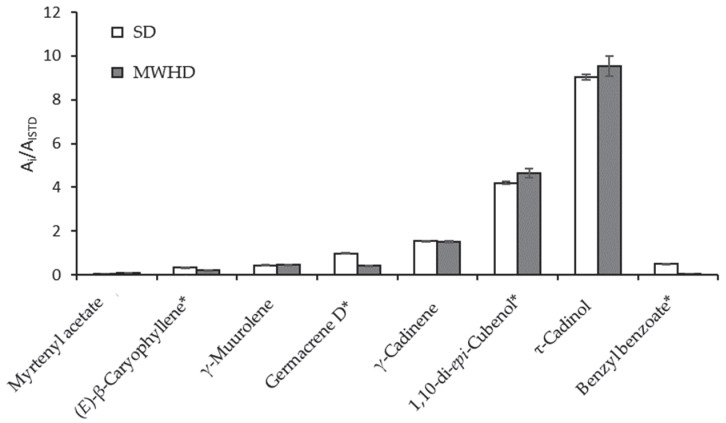
Comparison of major compound amounts in the EOs distilled from *S. aratocensis* via SD or MWHD. A_i_—GC chromatographic area of the substance; A_ISTD_—internal standard GC chromatographic area (*n*-tetradecane, 0.5 g L^−1^). * Values of A_i_/A_ISTD_ (GC/FID) with significant differences according to Student’s *t* test (*p* < 0.05).

Figure 3 shows the relative amount variation as a function of the employed distillation technique of the eight major *S. aratocensis* EO components measured as the GC/FID area relative to that of the internal standard (*n*-tetradecane). The relative amount of τ-cadinol found in the *S*. *aratocensis* EO under study (45–49%) was two time higher than that reported by Bueno et al. [6] (20%). The A_i_/A_ISTD_ ratio (Figure 3) showed that there was no significant difference in the τ-cadinol amount in the EOs obtained via SD or MWHD. The A_i_/A_ISTD_ ratios of (*E*)-β-caryophyllene, germacrene D, and benzyl benzoate were higher in the SD-EO than in the MWHD-EO. However, 1,10-di *epi*-cubenol was higher in the MWHD-EO (Figure 3).

### 2.3. Extract Chemical Characterization

**Figure 4:**
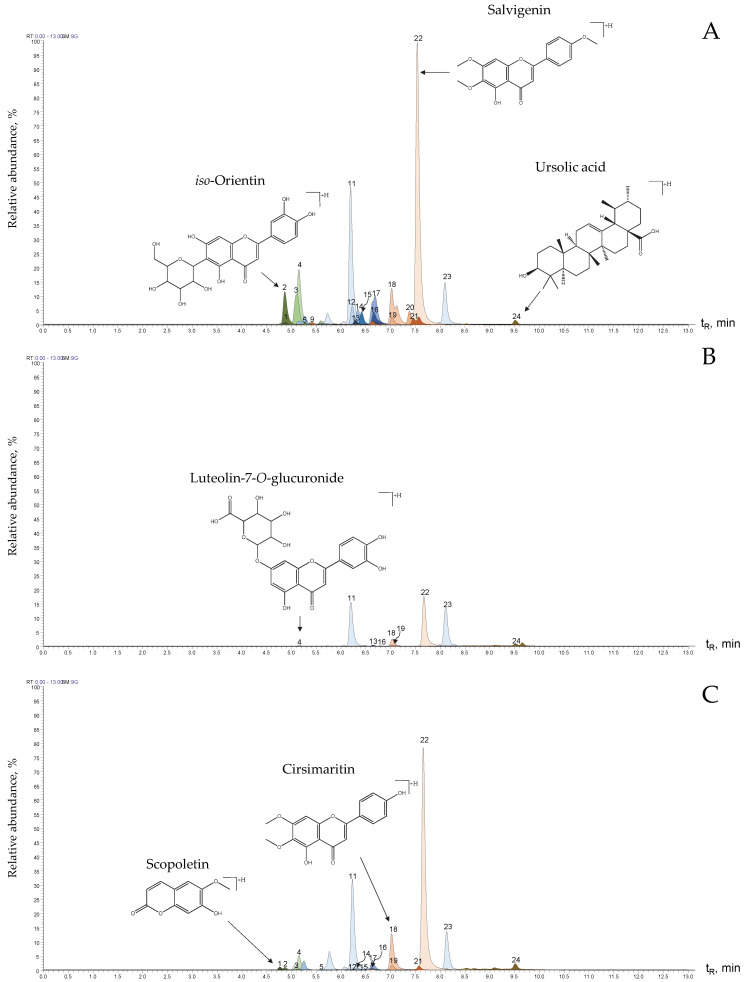
Extracted ion currents (EICs) of [M+H]^+^ ions (obtained using UHPLC-ESI^(+)^-Orbitrap-HRMS) from protonated molecules present in the *S. aratocensis* extracts isolated from (**A**) dry plant material before distillation; (**B**) dry residual plant material after distillation via SD; and (**C**) dry residual plant material after distillation via MWHD. See peak identification in Table 3.

*S*. *aratocensis* extracts were analyzed using UHPLC-ESI^(+/−)^-Orbitrap-HRMS operated in dual-positive and negative ion acquisition mode. The exact masses (*m*/*z*) of the protonated [M+H]^+^ or deprotonated [M−H]^−^ molecules of the substances in the *S*. *aratocensis* extracts, together with the exact masses and elemental formulas of the characteristic ions, are shown in Table 3. The extracted ionic currents (EICs) of the protonated molecules [M+H]^+^ of the substances in the extracts obtained from the plant material before and after distillation are shown in Figure 4.

A total of 21 compounds were identified in the *S*. *aratocensis* extract from the dry plant material before distillation. Standard substances allowed for the confirmatory identification of 14 compounds. A comparison of exact masses, the fragmentation patterns’ isotopic ratios of characteristic ions, and retention times (elution order) with those reported in the scientific literature led to presumptive identifications [9,10,18,19,20,21,22,23,24,25,26].

The presumptive LC/MS identification was conducted in two stages. First, the exact masses detected in full-scan mode and their corresponding elemental formulas were used to perform a search in databases such as PUBCHEM [23], FOODB [24], and Phenol-Explorer [27] to obtain a list of possible flavonoid-type molecules. The [M+H]^+^ and [M−H]^−^ ions were fragmented in the higher-energy dissociation chamber (HCD) to obtain their mass spectra at 10, 20,30, or 40 eV.

In the second stage, selected ion monitoring (SIM) was performed on those protonated or deprotonated molecules detected in the full scan; these ions were filtered by the quadrupole and stored in the C-trap, from whence they were sent to the HCD. The use of the quadrupole filter to isolate the ions of interest allowed for “cleaner” mass spectra and the execution of the quantification in a more exact and reproducible manner because, depending on the acquisition mode, several substances may eventually coelute and generate ions from different protonated or deprotonated molecules.

**Table 3:** Exact masses of protonated [M+H]^+^ and deprotonated [M−H]^−^ molecules and characteristic product ions in the mass spectra of the compounds identified via UHPLC-ESI^(+/−)^-Orbitrap-HRMS in *S. aratocensis* extracts.

Peak N° Figure 4	Compound	Formula	Exact Experimental Mass, *m*/*z*	∆ ppm	HCD, eV	Product Ions	Formula	*m*/*z,* I (%)	Identification Criteria	References
1	Scopoletin	C_10_H_8_O_4_	[M+H]^+^, 193.04939	0.78	20	[(M+H)-CH_3_]^+•^	C_9_H_6_O_4_	178.02588 (5%)	a, b, c	
2	*iso*-Orientin	C_21_H_20_O_11_	[M+H]^+^, 449.10739	1	30	[(M+H)-H_2_O]^+^	C_21_H_19_O_10_	431.09753 (42%)	a, b	[18]
						[(M+H)-2H_2_O]^+^	C_21_H_17_O_9_	413.08694 (64%)		
						[(M+H)-3H_2_O]^+^	C_21_H_15_O_8_	395.07559 (25%)		
						[(M+H)-C_3_H_6_O_3_]^+^	C_18_H_15_O_8_	359.07562 (15%)		
						[(M+H)-2H_2_O-C_2_H_4_O_2_]^+^	C_19_H_13_O_7_	353.06500 (30%)		
						[(M+H)-C_4_H_8_O_4_]^+^	C_17_H_13_O_7_	329.06589 (100%)		
						[(M+H)-C_5_H_10_O_5_]^+^	C_16_H_11_O_6_	299.05457 (38%)		
			[M−H]^−^, 447.09341	1	40	[(M−H)-C_3_H_6_O_3_]^−^	C_18_H_13_O_8_	357.06131 (15%)		
						[(M−H)-C_4_H_8_O_4_]^−^	C_17_H_11_O_7_	327.05112 (100%)		
						[(M−H)-C_5_H_8_O_5_]^−^	C_16_H_11_O_6_	299.05576 (37%)		
3	Vitexin	C_21_H_20_O_10_	[M+H]^+^, 433.11313	0.2	30	[(M+H)-H_2_O]^+^	C_21_H_19_O_9_	415.10272 (47%)	a, b, c	[21]
						[(M+H)-2H_2_O]^+^	C_21_H_17_O_8_	397.09216 (69%)		
						[(M+H)-C_4_H_8_O_4_]^+^	C_17_H_13_O_6_	313.07089 (100%)		
						[(M+H)-C_5_H_10_O_5_]^+^	C_16_H_11_O_5_	283.05991 (40%)		
			[M−H]^−^, 431.09833	0.1	20	[(M−H)-C_3_H_6_O_3_]^−^	C_18_H_13_O_7_	341.06644 (9%)		
						[(M−H)-C_4_H_8_O_4_]^−^	C_17_H_11_O_6_	311.05588 (100%)		
						[(M−H)-C_5_H_8_O_5_]^−^	C_16_H_11_O_5_	283.06094 (18%)		
4	Luteolin-7*-O-*glucuronide	C_21_H_18_O_12_	[M+H]^+^, 463.08655	1.19	10	[(M+H)-C_6_H_8_O_6_]^+^	C_15_H_11_O_6_	287.05501 (34%)	a, b, c	[20]
			[M−H]^−^, 461.07275	0.4	10	[(M−H)-C_6_H_8_O_6_]^−^	C_15_H_9_O_6_	285.04047 (25%)		
5	Luteolin-7-*O*-glucoside	C_21_H_20_O_11_	[M+H]^+^, 449.10730	1	10	[(M+H)-C_6_H_10_O_5_]^+^	C_15_H_11_O_6_	287.05460 (74%)	a, b, c	[9,19,20,21,22]
			[M−H]^−^, 447.09332	1	10	[(M−H)-C_6_H_10_O_5_]^−^	C_15_H_9_O_6_	285.04047 (15%)		
6	Salvianolic acid A	C_26_H_22_O_10_	[M−H]^−^, 493.11432	0.6	20	[(M−H)-C_9_H_10_O_5_]^−^	C_17_H_11_O_5_	295.06122 (14%)	a, b	[23,24,25]
						[(M−H)-C_15_H_16_O_7_]^−^	C_11_H_5_O_3_	185.02362 (37%)		
7	Salvianolic acid B	C_36_H_30_O_16_	[M−H]^−^, 717.14611	0.6	20	[(M−H)-C_9_H_8_O_4_]^−^	C_27_H_21_O_12_	537.10437 (43%)	a, b	[21,23,24,25,26]
						[(M−H)-C_9_H_10_O_5_]^−^	C_27_H_19_O_11_	519.09265 (36%)		
						[(M−H)-C_18_H_14_O_8_]^−^	C_18_H_15_O_8_	359.07706 (100%)		
						[(M−H)-C_18_H_20_O_10_]^−^	C_18_H_9_O_6_	321.04056 (37%)		
						[(M−H)-C_19_H_18_O_11_]^−^	C_17_H_11_O_5_	295.06061 (34%)		
8	Rutin	C_27_H_30_O_16_	[M+H]^+^, 611.16034	0.5	20	[(M+H)-C_6_H_10_O_4_]^+^	C_21_H_21_O_12_	465.10254 (5%)	a, b, c	[11,21]
						[(M+H)-C_6_H_10_O_4_-C_6_H_10_O_5_]^+^	C_15_H_11_O_7_	303.04941 (100%)		
			[M−H]^−^, 609.14673	0.5	20	[(M−H)-C_6_H_10_O_4_]^−^	C_21_H_19_O_12_	463.08844 (16%)		
						[(M−H)-C_12_H_20_O_9_]^−^	C_15_H_9_O_7_	301.03513 (12%)		
9	Quercetin-3-*O*-glucoside	C_21_H_20_O_12_	[M+H]^+^, 465.10236	0.9	30	[(M+H)-C_6_H_10_O_5_]^+^	C_15_H_11_O_7_	303.04941 (100%)	a, b, c	[9,22]
			[M−H]^−^, 463.08829	0.2	20	[(M−H)-C_6_H_10_O_5_]^−^	C_15_H_9_O_7_	301.03513 (12%)		
10	Rosmarinic acid	C_18_H_16_O_8_	[M+H]^+^, 361.09143	1	10	[(M+H)-C_9_H_10_O_5_]^+^	C_9_H_7_O_3_	163.03868 (26%)	a, b, c	[19,21,22]
			[M−H]^−^, 359.07736	0.3	10	[(M−H)-C_9_H_10_O_5_]^−^	C_9_H_5_O_3_	161.02362 (100%)		
11	N.I. (Figure S3)	C_20_H_22_O_5_	[M+H]^+^, 343.15353	1.3	20	[(M+H)-H_2_O]^+^	C_20_H_21_O_4_	325.14294 (69%)		
						[(M+H)-H_2_O-CO]^+^	C_19_H_21_O_3_	297.14810 (47%)		
						[(M+H)-H_2_O-CO-H_2_O]^+^	C_19_H_19_O_2_	279.13757 (20%)		
12	Naringenin	C_15_H_12_O_5_	[M+H]^+^, 273.07553	0.8	20	[(M+H)-C_8_H_8_O]^+^	C_7_H_5_O_4_	153.0181 (100%)	a, b, c	[19]
			[M−H]^−^, 271.06134	0.5	20	[(M−H)-C_8_H_8_O]^−^	C_7_H_3_O_4_	151.00285 (70%)		
						[(M−H)-C_7_H_4_O_4_]^−^	C_8_H_7_O	119.04929 (56%)		
13	Methyl-luteolin	C_16_H_12_O_6_	[M+H]^+^, 301.07089	0.76	30	[(M+H)-CH_3_]^+•^	C_15_H_10_O_6_	286.04736 (99%)	a, b	[10]
						[(M+H)-C_9_H_8_O_2_]^+^	C_7_H_5_O_4_	153.01846 (20%)		
			[M−H]^−^, 299.05609	0.06	20	[(M−H)-CH_3_]^−•^	C_15_H_8_O_6_	284.03287 (99%)		
14	Luteolin	C_15_H_10_O_6_	[M+H]^+^, 287.05478	0.82	30	[(M+H)-C_8_H_6_O_2_]^+^	C_7_H_5_O_4_	153.01837(38%)	a, b, c	[10,11]
			[M−H]^−^, 285.04046	0.3	30	[(M−H)-C_5_H_2_O_3_]^−^	C_10_H_7_O_3_	175.03937 (11%)		
						[(M−H)-C_8_H_6_O_2_]^−^	C_7_H_3_O_4_	151.00281 (20%)		
						[(M−H)-C_7_H_4_O_4_]^−^	C_8_H_5_O_2_	133.02858 (72%)		
15	N.I. (Figure S4)	C_31_H_26_O_15_	[M+H]^+^, 639.13336	1.69	20	[(M+H)-H_2_O]^+^	C_31_H_25_O_14_	621.12427 (63%)		
						[(M+H)-2H_2_O]^+^	C_31_H_23_O_13_	603.11332 (17%)		
						[(M+H)-C_14_H_14_O_8_]^+^	C_17_H_13_O_7_	329.06497 (79%)		
						[(M+H)-C_15_H_16_O_9_]^+^	C_16_H_11_O_6_	299.05444 (7%)		
						[(M+H)-C_21_H_20_O_11_]^+^	C_10_H_7_O_4_	191.03363 (40%)		
			[M−H]^−^, 637.11989	0.35	20	[(M−H)-C_10_H_8_O_5_]^−^	C_21_H_17_O_10_	429.08243 (9%)		
						[(M−H)-C_13_H_12_O_7_]^−^	C_18_H_13_O_8_	357.06137 (83%)		
						[(M−H)-C_14_H_14_O_8_]^−^	C_17_H_11_O_7_	327.05075 (100%)		
						[(M−H)-C_15_H_16_O_9_]^−^	C_16_H_9_O_6_	297.04013 (3%)		
16	Apigenin	C_15_H_10_O_5_	[M+H]^+^, 271.06030	0.74	30	[(M+H)-C_8_H_6_O]^+^	C_7_H_5_O_4_	153.01848 (36%)	a, b, c	[10]
			[M−H]^−^, 269.04565	0.4	30	[(M−H)-C_8_H_6_O]^−^	C_7_H_3_O_4_	151.00281 (8%)		
						[(M−H)-C_7_H_4_O_4_]^−^	C_8_H_5_O	117.03355 (50%)		
17	Jaceosidin	C_17_H_14_O_7_	[M+H]^+^, 331.08122	0.06	30	[(M+H)-CH_3_]^+•^	C_16_H_12_O_7_	316.05811 (22%)	a, b	[10]
						[(M+H)-CH_3_-H_2_O]^+•^	C_16_H_10_O_6_	298.04739 (100%)		
						[(M+H)-C_9_H_8_O_2_]^+^	C_8_H_7_O_5_	183.02911 (10%)		
			[M−H]^−^, 329.06680	0.38	20	[(M−H)-CH_3_]^−•^	C_16_H_10_O_7_	314.04330 (32%)		
						[(M−H)-CH_3_-CH_3_]^−^	C_15_H_7_O_7_	299.01984 (36%)		
18	Cirsimaritin	C_17_H_14_O_6_	[M+H]^+^, 315.08646	0.45	40	[(M+H)-CH_3_]^+•^	C_16_H_12_O_6_	300.06302 (5%)	a, b, c	[10,19,22,27]
						[(M+H)-CH_3_-H_2_O]^+•^	C_16_H_10_O_5_	282.05249 (5%)		
						[(M+H)-CH_3_-C_9_H_5_O_2_]^+•^	C_7_H_6_O_4_	154.02632 (31%)		
			[M−H]^−^, 313.07193	0.5	20	[(M−H)-CH_3_]^−•^	C_16_H_10_O_6_	298.04831 (13%)		
						[(M−H)-CH_3_-CH_3_]^−^	C_15_H_7_O_6_	283.02496 (23%)		
19	Eupatilin	C_18_H_16_O_7_	[M+H]^+^, 345.09686	0.57	30	[(M+H)-CH_3_]^+•^	C_17_H_14_O_7_	330.07269 (55%)	a, b, c	[10,24]
						[(M+H)-CH_3_-CH_3_]^+^	C_16_H_11_O_7_	315.04953 (<1%)		
			[M−H]^−^, 343.08237	0.11	30	[(M−H)-CH_3_]^−•^	C_17_H_12_O_7_	328.05847 (100%)		
						[(M−H)-CH_3_-CH_3_]^−^	C_16_H_9_O_7_	313.03516 (37%)		
20	Cirsilineol	C_18_H_16_O_7_	[M+H]^+^, 345.09686	0.05	30	[(M+H)-CH_3_]^+•^	C_17_H_14_O_7_	330.07278 (32%)	a, b	[24]
						[(M+H)-CH_3_-CH_3_]^+^	C_16_H_11_O_7_	315.04950 (16%)		
			[M−H]^−^, 343.08243	0.29	30	[(M−H)-CH_3_]^−•^	C_17_H_12_O_7_	328.05383 (84%)		
						[(M−H)-CH_3_-CH_3_]^−^	C_16_H_9_O_7_	313.03571 (5%)		
21	Acacetin	C_16_H_12_O_5_	[M+H]^+^, 285.07547	0.98	30	[(M+H)-CH_3_]^+•^	C_15_H_10_O_5_	270.05185 (21%)	b, c	[24]
						[(M+H)-CH_3_-CO]^+•^	C_14_H_10_O_4_	242.05701 (43%)		
			[M−H]^−^, 283.06199	0.03	20	[(M−H)-CH_3_]^−•^	C_15_H_8_O_5_	268.03662 (21%)		
22	Salvigenin	C_18_H_16_O_6_	[M+H]^+^, 329.10190	0.2	30	[(M+H)-CH_3_]^+•^	C_17_H_14_O_6_	314.07843 (17%)	b, c	
						[(M+H)-CH_3_-H_2_O]^+•^	C_17_H_12_O_5_	296.06790 (16%)		
						[(M+H)-CH_3_-H_2_O-CO]^+•^	C_16_H_12_O_4_	268.07309 (5%)		
						[(M+H)-CH_3_-H_2_O-2CO]^+•^	C_15_H_12_O_3_	240.07835 (100%)		
23	N.I. (Figure S5)	C_20_H_22_O_5_	[M+H]^+^, 343.15372	0.8	10	[(M+H)-C_3_H_8_]^+^	C_17_H_15_O_5_	299.09088 (15%)		
						[(M+H)-C_10_H_12_O_3_]^+^	C_10_H_11_O_2_	163.07530 (100%)		
						[(M+H)-C_10_H_12_O_3_-C_3_H_6_O]^+^	C_7_H_5_O	105.03375 (7%)		
24	Ursolic acid	C_30_H_48_O_3_	[M+H]^+^, 457.36713	1.1	10	[(M+H)-H_2_O]^+^	C_30_H_47_O_2_	439.35706 (48%)	b, c	[24]
			[M−H]^−^, 455.35333	0.6	-	-	-	-		

^a^ Tentative identification based on the measurement of the exact masses of the protonated [M+H]^+^ or deprotonated [M−H]^−^ molecules and their comparison with those reported for the genus *Salvia* [9,10,19,22,25,26]. ^b^ Tentative identification based on the study of the fragmentation pattern [ESI^(+/−)^—HRMS] and the isotopic ratio of the [M+H]^+^ and [M−H]^−^ ions and on the consultation of spectral databases [23,24]. ^c^ Confirmatory identification based on standard substances, i.e., scopoletin (≥98%), vitexin (≥95%), luteolin-7-*O*-glucuronide (≥98%), luteolin-7-*O*-glucoside (≥98%), rutin (≥94%), quercetin-3-*O*-glucoside (≥90%), rosmarinic acid (≥97%), naringenin (≥95%), luteolin (≥98%), apigenin (≥95%), cirsimaritin (≥98%), eupatilin (≥98%), acacetin (≥98%), salvigenin (≥98%), and ursolic acid (≥90%), via comparison of their mass spectra [ESI^(+/−)^—HRMS] and retention times (t_R_) with those of the substances present in the extracts. N.I.: not identified.

The mass spectrum of the *S*. *aratocensis* extract obtained from plant material before distillation contained a signal at *m*/*z* 449.10780 occurring at t_R_ = 4.82 min. This ion was filtered in SIM mode, and mass spectra were obtained in HCD at 10, 20, 30, and 40 eV. The mass spectra obtained in SIM mode (HCD, 30 eV) showed losses of three water molecules that gave rise to the product ions [(M+H)-H_2_O]^+^, [(M+H)-2H_2_O]^+^, and [(M+H)-3H_2_O]^+^, which appeared at *m*/*z* 431.08606, 413.08606, and 395.07559, respectively.

The loss of water from protonated flavonoid glycoside molecules may indicate the existence of a C-glycoside [28]. Considering the exact masses of the protonated [M+H]^+^ molecules and those of the product ions, the compound was initially identified as luteolin-C-hexoside. If the sugar was attached at the C-8 carbon, the molecule would be orientin, but if it was at the C-6 position, it would be *iso*-orientin.

**Figure 5:**
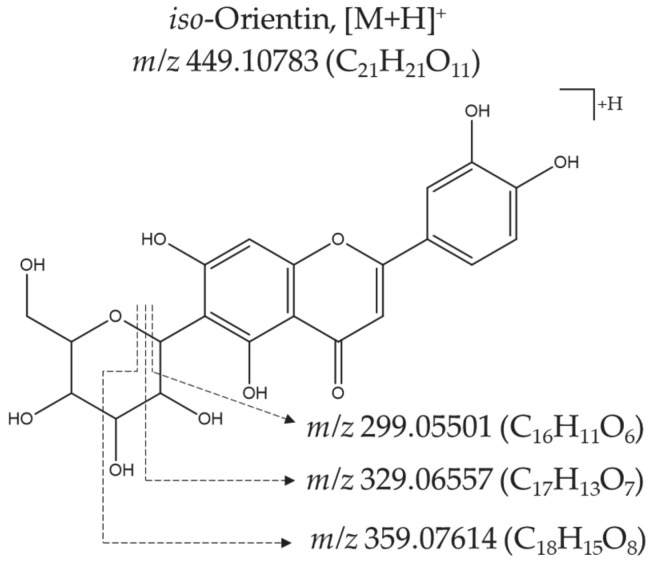
Formation of main product ions in the *iso*-orientin (luteolin-6-*C*-hexoside) mass spectrum obtained using ESI^(+)^-*Orbitrap*-HRMS (HCD, 30 eV).

Li et al. [18] showed that during the fragmentation of *iso*-orientin via the accelerated atom bombardment (FAB) method, the ion [(M+H)-3H_2_O]^+^ was detected with an abundance above 1%, while for orientin, the abundance of the same ion was below 1%. In the present work, the product ion [(M+H)-3H_2_O]^+^ was observed with a relatively high abundance (25%), which supports the identification of the compound as *iso*-orientin. Three fragments observed in the *iso*-orientin mass spectrum were products of sugar cleavages, as shown in Figure 5.

In the *iso*-orientin mass spectrum obtained using ESI^(−)^-MS, the product ions [(M−H)-C_3_H_6_O_3_]^−^ and [(M−H)-C_4_H_8_O_4_]^−^ were observed at *m*/*z* 357.016131 (15%) and at *m*/*z* 327.05112 (100%), respectively, and coincided with those reported by Chen et al. [29]. The product-ion [(M+H)-C_6_H_10_O_5_]^+^, corresponding to the aglycone luteolin (*m*/*z* 287.05501), was not detected in the *iso*-orientin (luteolin-6-*C*-hexoside) mass spectrum, which is contrary to what was observed in the luteolin-7-*O*-glucoside mass spectrum. The aglycone signal does appear in the latter at *m*/*z* 287.05460 (74%).

The parameters of the calibration curves used for the quantification of the *S*. *aratocensis* extract components are presented in Table 4. The substance amounts (expressed as mg of substance g^−1^ extract) in the *S*. *aratocensis* extracts obtained from plant material before or after distillation are shown in Table 5.

### 2.4. Antioxidant Activity

The antioxidant activities of the EOs and extracts of *S. aratocensis* measured using the ABTS^+•^ and ORAC methods are shown in Table 6.

## 3. Discussion

### 3.1. Salvia aratocensis Essential Oil Chemical Characterization

τ-Cadinol (44.4% and 48.8%) was the major component of the *S*. *aratocensis* EOs distilled via SD or MWHD, followed by 1,10-di-*epi*-cubenol (20.6% and 23.8%) and γ-cadinene (7.63% and 7.64%). τ-Cadinol is biosynthesized in plants via the mevalonic acid pathway in the cytosol of plant cells [30]. In τ-cadinol biosynthesis, farnesyl pyrophosphate binds to the active site of the cadinol synthase enzyme and, through multiple modifications, produces the cadinyl carbocation, which is stabilized by a hydroxylation reaction to form τ-cadinol [30,31]. τ-Cadinol has been found in low amounts in the EO of the aerial parts of other *Salvia* species, e.g., *S*. *sclarea* L. (0.1%) [32] and *S*. *verbenaca* L. (0.3–2.6%) [33], as well as in species of other genera, e.g., *Schinus molle* L. (21–29%), *Lobularia maritima* (L.) Desv. (3%) [34], *Psidium guajava* L. (1.3–6.4%) [35], *Lavandula angustifolia* Mill. [30], *Ocimum basilicum* L. (2.8–12.4%) [36,37,38], *Monticalia andicola* (Turcz.) C. Jeffrey (0.8%) [39], *Polyalthia suaveolens* Engl. & Diels (8.3%) [40], *Tanacetum* spp. (3.2%) [41], and *Siparuna guianensis* Aubl. (11.9–39.9%) [42].

The following biological activities have been reported for τ-cadinol: spasmolytic activity, according to a test incorporating guinea pig ileum [43]; muscle relaxant via calcium antagonist, according to investigations employing rat aorta [44]; bactericidal, as determined in tests with *Staphylococcus aureus*; and fungicidal activity, as determined in trials with *Trichophyton mentagrophytes* Robin [45]. τ-Cadinol regulates dendritic cells’ differentiation from human monocytes, which may have interesting applications for cancer treatment [46]. Due to its τ-cadinol content, *S*. *aratocensis* EO has great potential for use as a natural ingredient of phytopharmaceutical products. Additional experiments are necessary to increase EO distillation yields and τ-cadinol content.

### 3.2. Salvia aratocensis Extract’s Chemical Characterization

The identified *S*. *aratocensis* extract components include 13 flavones (vitexin, iso-orientin, luteolin-7-*O*-glucuronide, luteolin-7-*O*-glucoside, methyl-luteolin, luteolin, apigenin, jaceosidin, cirsimaritin, eupatilin, cirsilineol, acacetin, and salvigenin), 2 flavonols (rutin and quercetin-3-*O*-glucoside), 1 flavone (naringenin), 1 triterpene (ursolic acid), 3 phenolic acids (rosmarinic acid and salvianolic acids A and B), and 1 coumarin (scopoletin).

Ursolic acid, a pentacyclic triterpene, was the most abundant compound identified in the extract from dry plant material before distillation (37 ± 3 mg g^−1^). Its concentrations in the extracts from dry residual biomass after SD or MWHD were 28.9 ± 0.6 mg g^−1^ and 39.8 ± 0.6 mg g^−1^. Thus, SD resulted in a 22% decrease in ursolic acid content in the extract, while hydrodistillation had no significant effect.

Salvigenin, a compound that is more polar than ursolic acid, had larger content variations. The extract from dry plant material before distillation had a salvigenin content of 0.8 ± 0.1 mg g^−1^, while the post-distillation extracts had decreases of 67% (SD) and 46% (MWHD). The extract composition analysis showed that the post-distillation changes of low-polarity compounds were smaller than those of phenolic compounds. The phenolic compound losses may be a combination of thermal decomposition and their dissolution into the hydrosol, which is typically discarded. The substance amount decreases were greater in the residual biomass after SD than after MWHD.

The hydroalcoholic extraction yield from dry *S*. *aratocensis* plants before distillation was 19%. This means that approximately 703 mg of ursolic acid can be obtained from 100 g of this plant material. The ursolic acid amount would be reduced to 133 or 167 mg if plant residue after SD or MWHD was employed. It is possible to obtain four *S*. *aratocensis* harvests per year and 280 ± 38 g (aerial parts) of each harvested plant, offering a 64% moisture content. This translates into the production of approximately 2.8 g of ursolic acid per plant every year.

Ursolic acid is present in a large number of Lamiaceae species [47]. It has important biological properties, including cytotoxic activity against HL-60, BGC, BEL-7402, and HeLa cancer lines [48]. It has anti-inflammatory activity, according to studies concerning the enzymes involved in the inflammatory cascade [49]. Additionally, it is an apoptosis inducer, according to results obtained with A431 squamous cell carcinoma model cell lines and those derived from HaCat keratinocytes [50]. It is also an active compound against *Mycobacterium tuberculosis* [51]. It inhibits cholesterol synthesis, according to in vivo studies with mice [52]. The DPPH^•^ assay revealed its antiradical activity [53]. A review described other biological activities of ursolic acid, extraction methods, and a collection of patents on its uses in cosmetics (49) and pharmaceuticals (97) [47].

The flavones identified in the *S*. *aratocensis* extracts, i.e., apigenin, cirsimaritin, jaceosidin, eupatilin, luteolin, and their glycosylated derivatives, have been found in other species of the same genus, e.g., *S*. *officinalis* [9], *S*. *plebeia* R. Br. [10], and *S*. *nemorosa* L. [11]. Luteolin-7-*O*-glucuronide was the most abundant flavone in the *S*. *aratocensis* extract from pre-distillation plant material (25.3 ± 0.3 mg g^−1^ extract). Dapkevicius et al. [54] studied the antiradical activity of a *Thymus vulgaris* L. extract using a DPPH online HPLC method and found that luteolin-7-*O*-glucuronide was active. In vitro assays employing human lymphocytes that were conducted by Orhan et al. [55] showed that luteolin-7-*O*-glucuronide at 40 μM was not toxic. These authors found that it has great potential as an antigenotoxic agent against aflatoxin B1. Based on in vitro cellular assays, Cho et al. [56] demonstrated that luteolin-7-O-glucuronide exhibits anti-inflammatory and antioxidant properties.

### 3.3. Antioxidant Activity of Essential Oil and Extracts

The extract from dry plant material before distillation showed the highest antioxidant activity among the samples examined with the ABTS^+•^ assay. *S*. *aratocensis* EOs, and the extract from dry plant material before distillation, had about twice the ORAC antioxidant activity of the extract from the post-SD residual biomass. The distillation technique had a small effect on the Eos’ antioxidant activity (1520 ± 9 μmol Trolox^®^ g^−1^ for SD; 1610 ± 67 μmol Trolox^®^ g^−1^ for MWHD). The lower number of phenolic compounds in the SD residual biomass was consistent with the reduced antioxidant activity of its extract. The measured antioxidant activities of *S*. *aratocencis* EOs and extracts were higher than those of commercial antioxidants such as BHT (98 ± 5 μmol Trolox^®^ g^−1^) and α-tocopherol (450 ± 50 μmol Trolox^®^ g^−1^).

## 4. Materials and Methods

### 4.1. Reagents

Trolox^®^, α-tocopherol, butylated hydroxytoluene (BHT), 2,2′-azino-bis(3-ethylbenzothiazoline-6-sulfonic acid) (ABTS), 2,2′-azobis(2-amidinopropane) dihydrochloride (AAPH), fluorescein, sodium persulfate, sodium acetate, *n*-tetradecane (≥99%), quercetin-3-*O*-glucoside (≥90%), rosmarinic acid (≥97%), naringenin (≥95%), apigenin (≥95%), eupatilin (≥98%), ursolic acid (≥90%), rutin (≥94%), (*E*)-β-caryophyllene (≥98.5%), α-humulene (≥96%), (2*E*, 6*Z*)-farnesol (≥96%), benzyl benzoate (≥99%), and benzyl salicylate (≥99%) were acquired from Sigma-Aldrich (St. Louis, MO, USA). Dipotassium phosphate was obtained from J.T. Baker (Phillipsburg, NJ, USA). Luteolin-7-*O*-glucoside (≥98%), luteolin-7-*O*-glucuronide (≥98%), cirsimaritin (≥98%), acacetin (≥98%), scopoletin (≥98%), and salvigenin (≥98%) were acquired from ChemFaces (Wuhan, China). Vitexin (≥95%) was obtained from Phytolab (Vestenbergsgreuth, Germany). Luteolin (≥98%) was purchased from Chemcruz (Santa Cruz Biotech., Dallas, TX, USA). The mixture of *n*-alkanes C_8_-C_25_ was obtained from AccuStandard, Inc. (New Haven, CT, USA). Helium, air, hydrogen, and nitrogen (99.995%) for GC analysis were purchased from *Messer* (Bucaramanga, Colombia). Ethanol (96%) was acquired from Suquin S.A.S. (Bucaramanga, Colombia). Type I water (18.2 MΩ cm) was produced using a Millipore Direct-QTM (Merck, Darmstadt, Germany) purification system.

### 4.2. Plant Material

*S. aratocensis* was cultivated—from cuttings collected in the field—at the CENIVAM Research Center located on the central campus of Universidad Industrial de Santander, Bucaramanga, Santander, Colombia (07°08.422′ N 073°06.960′ W). Botanical identification was carried out in the National Herbarium of the Institute of Natural Sciences of the National University of Colombia, Bogotá, Colombia (voucher number COL517740). The *S. aratocensis* plants collected for distillation and extraction were in a flowering stage and only their aerial undamaged parts were used. The collection permit for gathering *S. aratocensis* in Chicamocha Canyon (control sheets for herbarium and cuttings for cultivation) was obtained through the contract for access to genetic resources and derived products at N° 270 signed between Universidad Industrial de Santander and the Ministry of Environment and Sustainable Development.

### 4.3. Essential Oil Distillation

EOs were distilled from fresh *S. aratocensis* plants via two methods: (1) hydrodistillation assisted by microwave radiation (MWHD) in a modified household microwave oven (Model MS32J5133AG, Samsung, Negerin Sembilan, Malaysia) according to the method reproted by Stashenko et al. [57] and (2) steam distillation (SD) using a 0.1 m^3^ stainless-steel distiller. Briefly, for MWHD distillation, freshly cut *S. aratocensis* aerial parts (350 g) were suspended in water (500 mL) in a 2 L flask attached to a Clevenger-type apparatus with a Dean–Stark distillation reservoir and spiral and spherical condensers. The aqueous mixture was subjected to microwave radiation for 1.5 h (15 min × 6). Regarding SD, the freshly cut plant material (19 kg) was compacted to a charge density of 316.3 kg m^−3^ in a 0.1 m^3^ still. Steam was generated in a TECNIK N-553 6 BHP boiler (Tecnik^®^ Ltd., Bogotá, Colombia), which was operated at 5 × 10^5^ Pa with a condensate flow of 180 mL min^−1^. Distillation was carried out for 3 h. The EOs were dried with anhydrous sodium sulfate and stored in a refrigerator at 4 °C until analysis and use.

### 4.4. Extraction

*S. aratocensis* extracts were obtained from dry plant material before and after distillation according to the method reported by Durling et al. [58] with some modifications. Briefly, the plant material (100 g) was deposited in a 4 L glass container, to which an aqueous solution of ethanol in water (2 L, 70% v v^−1^) was added. The extractions were carried out in an Elmasonic S15H ultrasonic bath (Elmasonic, Signen, Germany) (37 kHz) for 1 h at 50 °C. The extracts were filtered (Whatman N° 1 paper), concentrated in a Heidolph^®^ Basis Hel-Vap HL rotary evaporator (Schwabach, Germany), dried in a VirTis^®^ AdVantage Plus lyophilizer (New York, USA), and stored in amber glass containers under a nitrogen atmosphere until analysis.

### 4.5. GC/FID and GC/MS Essential Oil Analysis

The EOs of *S. aratocensis* were analyzed via GC/FID/MS in a gas chromatograph (GC 6890 System Plus, Agilent Technologies, AT, Palo Alto, CA, USA) with a mass-selective detector (AT, MSD 5973 Network) and a flame ionization detection system (FID 250 °C) using electron ionization (EI, 70 eV). Sample introduction (EO in CH_2_Cl_2_ at 1.2%) was performed using an automatic injector, which was operated in split mode (ratio 1:30), and the injection port temperature was 250 °C. Capillary DB-5 and DB-WAX (J & W Scientific, Folsom, CA, USA) columns with the same dimensions, namely, 60 m × 0.25 mm, I.D. × 0.25 µm, d_f_., were used. The initial pressure at the head of the column was 113 × 10^3^ Pa, and a constant flow (1.0 mL min^−1^) of the helium carrier gas (99.995%, Messer, Bucaramanga, Colombia) was maintained. The temperature of the chromatographic oven was programmed to increase from 50 °C (5 min) to 150 °C (2 min) at 5 °C/min and then to 230 °C (10 min) at 5 °C/min. When the DB-5 column was used, ramping with additional heating was employed (up to 275 °C (15 min) at 10 °C/min). The transfer line temperature in the GC/MS system was 285 °C. The mass range used for the acquisition in full-scan mode was *m*/*z* 40–450 u, with an acquisition speed of 3.58 scan s^−1^, which was set using the MSD ChemStation Ver. G1701DA AT software. Compound identification was based on the linear retention indices (LRIs) and by comparison of the experimental mass spectra with those reported in the Adams 2004 [16], NIST 2017 [15] and Wiley 2008 [17] databases. Standard substances were used, i.e., (*E*)-β-caryophyllene, α-humulene, (2*E*, 6*Z*)-farnesol, benzyl benzoate, and benzyl salicylate, which were analyzed under the same chromatographic conditions as the EOs.

### 4.6. UHPL-ESI^(+/−)^-Orbitrap-HRMS Analysis of the Extracts

The *S. aratocensis* hydroethanolic extracts were analyzed in a Vanquish^TM^ ultrahigh-performance liquid chromatograph (UHPLC, Thermo Scientific, Waltham, MA, USA), equipped with a degassing unit, a gradient binary pump, and an autosampler, kept at 10 °C. A Zorbax Eclipse XDB C_18_ column (Sigma Aldrich, St. Louis, MO, USA) of 50 mm L × 2.1 mm I.D. and a 1.8 µm particle size was used. The column compartment was kept at 40 °C. The flow rate of the mobile phase containing Type I water (A) and MeOH (B), both incorporating formic acid (0.1%) and ammonium formate (5 mM), was 300 μL/min. The initial gradient condition was 100% A, which was changed linearly to 100% B after 8 min, held for 4 min, returned to 100% A after 1 min, and then held for 3 min. The injection volume was 2 μL. The UHPLC was connected to a Q-Exactive Plus Orbitrap mass spectrometer (Thermo Scientific, Bremen, Germany) with a heated electrospray ionization source (HESI-II) and polarity exchangers for periods <500 ms with fragmentation at the HCD. The capillary voltage was 3.5 kV. The nebulizer and capillary temperatures were 350 °C and 320 °C, respectively. Nitrogen (>99% purity) was obtained from a nitrogen generator (Peak Scientific, Scotland, UK). The sheath gas and auxiliary gas (N_2_) were set at 40 and 10 arbitrary units, respectively. During the full scan of the MS, the mass resolution was set at 70,000 (full width at half maximum (at *m*/*z* 200)—FWHM) with an automatic gain control target of 3 × 10^6^, a maximum injection time of the C-trap of 200 ms, and a mass range of *m*/*z* 80–1000. Ions injected into the higher-energy dissociation chamber (HCD) via the C-trap were fragmented with normalized collision energies through steps from 10 to 70 eV. Mass spectra were recorded in the all-ion fragmentation mode for each collision energy, employing a mass resolution of 35,000. Full instrument calibration was performed every fortnight using Pierce LTQ Velos ESI positive ion calibration solution and a Pierce ESI negative ion calibration solution (Thermo Scientific, Rockford, IL, USA). Data were analyzed using Thermo Xcalibur 3.1 software (Thermo Scientific, San José, CA, USA).

### 4.7. Antioxidant Activity

#### 4.7.1. ABTS^+•^ Assay

The in vitro antioxidant activity of the *S. aratocensis* EOs and extracts was evaluated using the ABTS^+•^ assay, which was performed according to the methodology described by Re et al. [59] with some modifications. In brief, in an aqueous sodium acetate solution (50 mL, 20 mM, and pH = 4.5), ABTS (7 mM) and potassium persulfate (PDS) (2.45 mM) were reacted for 24 h in the absence of light to produce the radical cation ABTS^+•^. Absorbance readings were taken at a wavelength corresponding to λ = 750 nm and at a temperature of 25 °C. Antioxidant activity was expressed as μmol Trolox^®^. All measurements were performed in triplicate, and the results were expressed as the mean ± standard deviation.

#### 4.7.2. ORAC Assay

The in vitro antioxidant activity of the *S. aratocensis* EOs and extracts was measured using the ORAC assay, which was performed according to the procedure described by Huang et al. [60] with some modifications. A Varioskan LUX VL0000D0 spectrophotometer (Thermo Scientific, Singapore), equipped with 200 µL 96-well poly(styrene) black microplates, was used under the fluorescence module. Diluted samples of the EOs and extracts (25 µL) were added to each well, and a fluorescein solution (150 µL and 8.16 × 10 ^−5^ mM in phosphate buffer) was added. The mixture was incubated for 18 min at 37 °C and was completed with an AAPH solution (25 µL, 153 mM, in phosphate buffer). Fluorescence was measured for 80 min with excitation wavelengths of λ = 490 nm and emission wavelengths of λ = 520 nm. The antioxidant capacity was determined according to the difference between the area under the curve of the sample and the blank of the phosphate buffer reaction. All measurements were performed in triplicate, and the results were expressed as the mean ± standard deviation.

## 5. Conclusions

Both steam distillation and hydrodistillation assisted by microwave radiation afforded relatively low yields (*ca.* 0.1%) of *S*. *aratocensis* EO. The major EO compounds were the sesquiterpenols τ-cadinol (44–48%) and 1,10-di-*epi*-cubenol (21–24%). The EO exhibited antioxidant activity (1520–1610 μmol Trolox^®^ g^−1^), measured using the ORAC method, that was higher than the reference substances BHT (98 μmol Trolox^®^ g^−1^) and α-tocopherol (450 μmol Trolox^®^ g^−1^). The *S. aratocensis* extract obtained from dry plant material before distillation (extraction yield 19%) was rich in ursolic acid (37 ± 3 mg g^−1^) and luteolin-7-*O*-glucuronide (25.3 ± 0.3 mg g^−1^). This extract’s antioxidant activity, which was measured using the ORAC method, was high (1300 ± 14 μmol Trolox^®^ g^−1^). The antioxidant activities of the extracts obtained from dry plant material after SD (720 ± 75 μmol Trolox^®^ g^−1^) or MWHD (1200 ± 12 μmol Trolox^®^ g^−1^) were lower than those of the extract isolated from dry plant material before distillation but higher than those of the reference substances (BHT and α-tocopherol). These results show that *S. aratocensis*, a fast-growing, resistant, and easy-to-grow native plant, is amenable to the application of circular economic approaches; that is, its essential oil, which is rich in τ-cadinol, and its extract can be obtained from residual biomass rich in ursolic acid, with both products possessing high antioxidant activity.

## Figures and Tables

**Table 1:** Yields (%) of the essential oils distilled from *S. aratocensis* via SD and MWHD and those of the hydroethanolic extracts.

Essential Oil Yield, %		Extract Yield, %		
SD	MWHD	Dry Material before Distillation	Dry Residual Material after Distillation	
			SD	MWHD
0.07	0.08	19	4.6	4.2

**Table 2:** Chemical characterization via GC/FID and GC/MS of the EOs distilled from *S. aratocensis* plants via steam distillation and hydrodistillation assisted by microwave radiation.

Peak N° Figure 2	Compound	Linear Retention Indices				Relative GC/FID Area (DB-5 Column), %					
		DB-5		DB-WAX							
		Exp.	Lit.	Exp.	Lit.	SD (±S, *n* = 3)			MWHD (±S, *n* = 3)		
1	Myrtenyl acetate ^a,b^	1325	1328 [14]	1691	1698 [15]	0.170	±	0.001	0.48	±	0.02
2	β-Elemene ^a,b^	1396	1390 [14]	1595	1590 [14]	0.202	±	0.007	0.215	±	0.007
3	(*E*)-β-Caryophyllene ^a,b,c^	1432	1420 [14]	1606	1598 [14]	1.60	±	0.04	1.13	±	0.03
4	(*Z*)-Muurola-3,5-diene ^a,b^	1456	1454 [15]	1645	1630 [15]	0.191	±	0.001	0.225	±	0.008
5	α-Humulene ^a,b,c^	1468	1472 [6]	1680	1666 [14]	0.726	±	0.002	0.61	±	0.01
6	γ-Muurolene ^a,b^	1474	1477 [15]	1696	1689 [14]	2.17	±	0.01	2.32	±	0.06
7	γ-Curcumene ^a,b^	1486	1490 [6]	1474	1474 [15]	0.716	±	0.006	0.54	±	0.03
8	Germacrene D ^a,b^	1493	1481 [6]	1718	1708 [14]	4.85	±	0.01	2.10	±	0.01
9	Valencene ^a,b^	1498	1496 [16]	1724	1728 [14]	0.442	±	0.001	0.56	±	0.02
10	α-Farnesene ^a,b^	1506	1504 [15]	1745	1743 [14]	1.861	±	0.006	-		
11	Germacrene A ^a,b^	1514	1518 [6]	1754	1743 [14]	0.520	±	0.001	0.50	±	0.01
12	γ-Cadinene ^a,b^	1525	1532 [6]	1767	1763 [14]	7.63	±	0.03	7.64	±	0.09
13	(*Z*)-Calemenene ^a,b^	1531	1537 [6]	1835	1834 [14]	0.728	±	0.008	0.92	±	0.01
14	(*Z*)-Muurol-5-en-4-β-ol ^a,b^	1541	1550 [16]	1974	-	0.466	±	0.003	0.511	±	0.002
15	α-Cadinene ^a,b^	1546	1550 [6]	1797	1815 [15]	0.608	±	0.005	0.56	±	0.01
16	Selina-3,7(11)-diene ^a,b^	1549	1545 [16]	1785	1792 [15]	-			-		
17	Elemol ^a,b^	1556	1548 [16]	2079	2078 [14]	0.116	±	0.001	0.217	±	0.004
18	Spirojatamol ^a,b^	1593	1592 [15]	-	-	0.275	±	0.009	0.318	±	0.001
19	Gleenol ^a,b^	1605	1610 [6]	2039	2051 [15]	0.296	±	0.003	0.326	±	0.002
20	1,10-di-*epi*-Cubenol ^a,b^	1631	1632 [6]	2067	2074 [14]	20.6	±	0.1	23.75	±	0.05
21	τ-Cadinol ^a,b^	1660	1667 [6]	2182	2169 [15]	44.4	±	0.3	48.8	±	0.3
22	C_15_H_24_O ^d^ (N.I., Figure S1)	1665	-	2337	-	1.56	±	0.02	2.06	±	0.02
23	α-Cadinol ^a,b^	1668	1677 [6]	2234	2227 [14]	0.82	±	0.05	0.64	±	0.08
24	C_15_H_24_O ^e^ (N.I., Figure S2)	1683	-	2377	-	1.10	±	0.01	1.14	±	0.01
25	α-Bisabolol ^a,b^	1694	1699 [6]	2217	2213 [14]	0.92	±	0.01	1.006	±	0.005
26	(2*E, 6Z*)-Farnesol ^a,b,c^	1718	1722 [15]	2353	2356 [15]	0.223	±	0.001	0.302	±	0.005
27	Benzyl benzoate ^a,b,c^	1780	1762 [15]	2648	2638 [14]	2.52	±	0.04	0.19	±	0.01
28	Farnesyl acetate ^a,b^	1834	1843 [15]	2260	2260 [15]	0.404	±	0.008	-		
29	Benzyl salicylate ^a,b,c^	1884	1869 [15]	-	2784 [15]	0.50	±	0.01	-		
30	Phytol ^a,b^	2107	2114 [15]	2620	2622 [15]	-			0.189	±	0.005

^a^ Tentative identification based on retention times (t_R_) and linear retention indices measured using DB-5 (nonpolar) and DB-WAX (polar) columns [6,14,15,16]. ^b^ Tentative identification based on mass spectra (MS; electron ionization, 70 eV, >95% coincidence), study of fragmentation patterns, and comparison with MS spectra from NIST (2017) [15], Adams (2007) [16], and Wiley (2008) [17] spectral databases. ^c^ Confirmatory identification based on standard substances, namely, (*E*)-β-caryophyllene (98.5%) (LRI_DB-5_ = 1434, LRI_DB-WAX_ = 1611), α-humulene (96%) (LRI_DB-5_ = 1470, LRI_DB-WAX_ = 1683), (2*E*, 6*Z*)-farnesol (96%) (LRI_DB-5_ = 1718, LRI_DB-WAX_ = 2361), benzyl benzoate (99%) (LRI_DB-5_ = 1780, LRI_DB-WAX_ = 2652), and benzyl salicylate (99%) (LRI_DB-5_ = 1884, LRI_DB-WAX_ = 2792), and via comparison of their mass spectra and retention times (t_R_) with those of the EO components. ^d^ Mass spectrum is shown in Figure S1 (Supplementary Materials). ^e^ Mass spectrum is shown in Figure S2 (Supplementary Materials). N.I.: not identified.

**Table 4:** Chromatographic parameters used for the quantitation of standard compounds and the confirmatory identification of *S. aratoncensis* extract constituents.

Standard Compound	Linear Dynamic Range, μg mg^−1^	Equation	R^2^	LOD, μg mg^−1^	LOQ, μg mg^−1^
Scopoletin	0.02–0.8	y = 463800386x + 2528726	0.998	0.04	0.13
Vitexin	0.06–0.8 ^1^	y = 29902795x − 234075	0.996	0.05	0.18
	1–10 ^2^	y = 23632604x + 16237131	0.993		
Luteolin-*7-O-*glucuronide	0.02–0.8 ^1^	y = 11017361x + 29367	0.997	0.04	0.15
	1–10 ^2^	y = 13477938x − 3948338	0.993		
Luteolin-7-*O*-glucoside	0.04–0.8	y = 30330792x + 943824	0.995	0.06	0.21
Rutin	0.06–0.8	y = 7754585x + 157454	0.992	0.07	0.23
Quercetin-3-*O*-glucoside	0.04–0.8	y = 12031321x − 113291	0.997	0.05	0.16
Rosmarinic acid	0.06–0.8	y = 31520429x − 1206359	0.991	0.08	0.28
Naringenin	0.06–0.8 ^1^	y = 29932955x + 344622	0.997	0.04	0.14
	0.6–6 ^2^	y = 18445875x+ 9232124	0.995		
Luteolin	0.04–0.8	y = 62182328x + 1738473	0.996	0.06	0.21
Apigenin	0.06–0.8	y = 68451624x + 3092256	0.994	0.08	0.26
Cirsimaritin	0.1–0.8	y = 426952447x + 16851979	0.990	0.09	0.30
Eupatilin	0.02–0.8	y = 197167110x − 1075471	0.997	0.04	0.13
Acacetin	0.04–0.8	y = 228671251x + 1965791	0.995	0.06	0.21
Salvigenin	0.06–0.8	y = 910743362x − 14489550	0.994	0.07	0.23
Ursolic acid	0.04–0.8	y = 852032x + 73796	0.995	0.06	0.20

^1^ Low concentration level. ^2^ High concentration level.

**Table 5:** Amounts of the substances identified in the extracts of *S. aratocensis*, obtained from dry plant material before distillation and from dry plant material after distillation, via SD or MWHD.

Compound	Amount, mg g_extract_ ^−1^ (±S, *n* = 3)		
	Plant Material		
	Dry, before Distillation	After Distillation	
		SD	MWHD
Scopoletin	<LOQ	<LOQ	<LOQ
Vitexin	3.07 ± 0.06	0.087 ± 0.007	0.35 ± 0.01
Luteolin-*7-O-*glucuronide	25.3 ± 0.3	1.16 ± 0.06	3.9 ± 0.2
Luteolin-7-*O*-glucoside	0.155 ± 0.003	<LOQ	<LOQ
Rutin	<LOQ	N.D.	N.D.
Quercetin-3-*O*-glucoside	0.3 ± 0.1	N.D.	N.D.
Rosmarinic acid	2.0 ± 0.4	<LOQ	0.73 ± 0.01
Naringenin	1.84 ± 0.08	<LOQ	<LOQ
Luteolin	0.70 ± 0.06	<LOQ	<LOQ
Apigenin	0.423 ± 0.002	<LOQ	<LOQ
Cirsimaritin	<LOQ	<LOQ	<LOQ
Eupatilin	0.27 ± 0.06	<LOQ	<LOQ
Acacetin	<LOQ	<LOQ	<LOQ
Salvigenin	0.8 ± 0.1	0.26 ± 0.08	0.43 ± 0.07
Ursolic acid	37 ± 3	28.9 ± 0.6	39.8 ± 0.6

N.D.: not detected.

**Table 6:** Antioxidant activity (measured using the ABTS^+•^ and ORAC methods) of the essential oils distilled from *S. aratocensis* via steam distillation (EO-SD) and via hydrodistillation assisted by microwave radiation (EO-MWHD) and of the *S. aratocensis* extracts isolated from dry plants and after their distillation via SD or MWHD.

Sample	Plant Material	Antioxidant Activity, μmol Trolox^®^ g^−1^ (±S, *n* = 3)	
		ABTS^+•^	ORAC
EO-SD	Fresh	49 ± 1	1520 ± 9
EO-MWHD	Fresh	32.1 ± 0.1	1610 ± 67
Extract	Before distillation (dried)	82 ± 4	1303 ± 14
	Biomass residue after SD (dried)	51 ± 4	720 ± 75
	Biomass residue after MWHD (dried)	73 ± 5	1205 ± 12
BHT		4990 ± 60	98 ± 5
α-Tocoferol		2310 ± 40	450 ± 50

## Data Availability

The supporting data are found in the database of the CIBIMOL research group, Universidad Industrial de Santander, Bucaramanga, Colombia.

## Supplementary Materials

The following supporting information can be downloaded at: https://www.mdpi.com/article/10.3390/molecules28104062/s1, Figure S1: Mass spectrum obtained by GC/MS (EI, 70 eV) a sesquiterpenoid (peak N° 22 in Table 2, LRI 1665), found in the *S. aratocensis* EO distilled by microwave-assisted hydrodistillation (MWHD); Figure S2: Mass spectrum obtained by GC/MS (EI, 70 eV) a sesquiterpenoid (peak N° 24 in Table 2, LRI 1683) in the *S. aratocensis* EO distilled by microwave-assisted hydrodistillation (MWHD); Figure S3: Mass spectrum obtained by UHPL-ESI^(+)^-Orbitrap-MS (SIM, *m/z* 343; HCD, 20 eV) of the compound N° 11 (Table 3) present in the hydroethanolic extract of *S. aratocensis* obtained from fresh plant before distillation; Figure S4: Mass spectrum obtained by UHPL-ESI^(+)^-Orbitrap-MS (SIM, *m/z* 639; HCD, 20 eV) of the compound N° 15 (Table 3) present in the hydroethanolic extract of *S. aratocensis* obtained from fresh plant before distillation; Figure S5: Mass spectrum obtained by UHPL-ESI^(+)^-Orbitrap-MS (SIM, *m/z* 343; HCD, 20 eV) of compound N° 23 (Table 3) present in the hydroethanolic extract of *S. aratocensis* obtained from vegetal material before distillation.

## Sample Availability

Samples of *S. aratocensis* EOs and extracts are available at CENIVAM—Industrial University of Santander (Bucaramanga, Colombia).

## Abbreviations

AT—Agilent Technologies; EI—electron ionization; EIC—extracted ion current; EO—essential oil; EO-SD—essential oil obtained via steam distillation; EO-MWHD—essential oil obtained using microwave-assisted hydrodistillation; ESI—electrospray ionization; eV—electron volt; FID—flame ionization detector; d_f_—film thickness; GC—gas chromatography; HCD—higher-energy collision dissociation cell; UHPLC—ultrahigh-performance liquid chromatography; HRMS—high-resolution mass spectrometry; I, %—intensity; I.D.—internal diameter; LC—liquid chromatography; LOD—limit of detection; LOQ—limit of quantification; LRI—linear retention index; MS—mass spectrometry or mass spectrum; *m*/*z*—mass-to-charge ratio; MWHD—microwave-assisted hydrodistillation; SD—steam distillation; t_R—_retention time.
