# Supplementary material for: Chemical Composition and In Vitro Antioxidant Activity of Salvia aratocensis (Lamiaceae) Essential Oils and Extracts
Source: Molecules. 2023 May 12;28(10):4062. doi: 10.3390/molecules28104062 (PMC10223537; doi:10.3390/molecules28104062)
Supplement: Supplementary file 1 [file molecules-28-04062-s001.zip › molecules-2364647-supplementary.pdf]

molecules

MDPI

Supplementary material

Chemical composition and *in vitro* antioxidant activity of *Salvia aratocensis* (Lamiaceae) essential oils and extracts

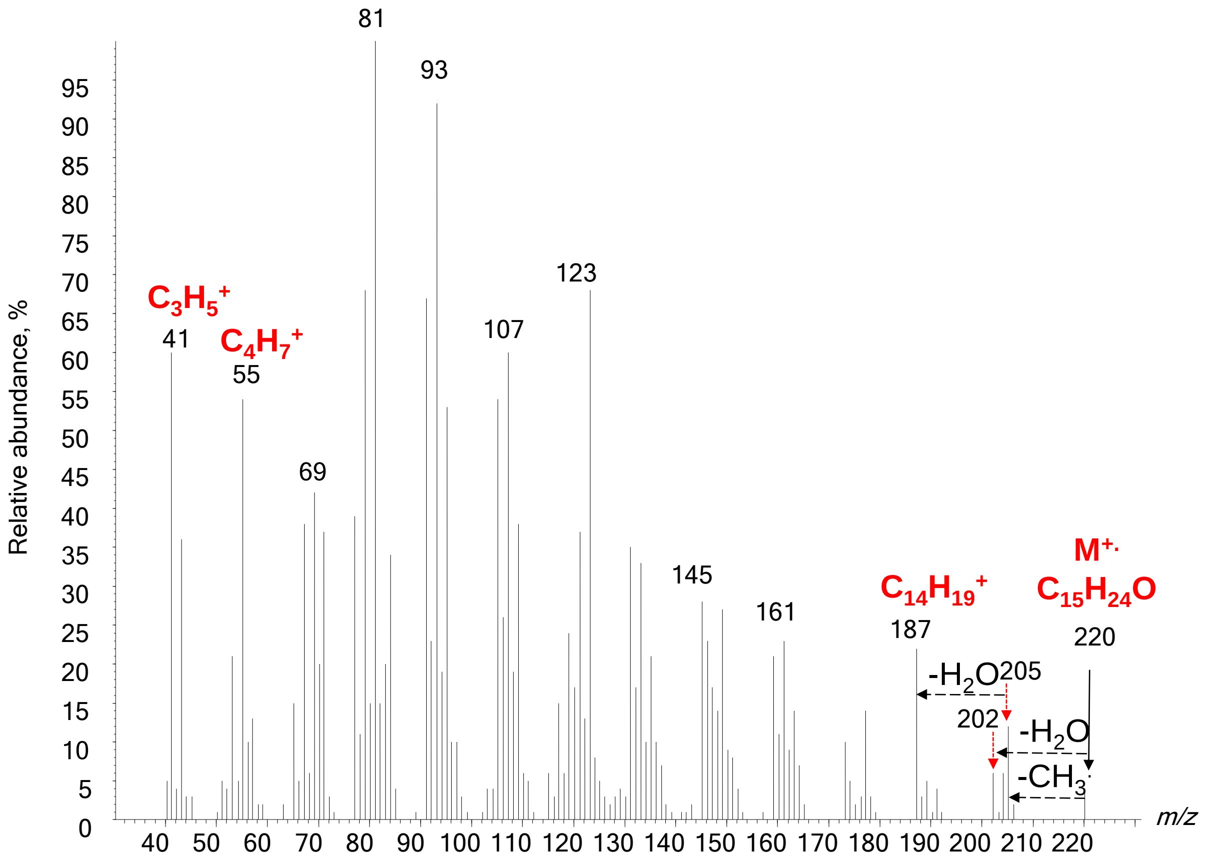

Figure S1. Mass spectrum obtained by GC/MS (EI, 70 eV) a sesquiterpenoid (peak N° 22 in **Table 2**, LRI 1665), found in the *S. aratocensis* EO distilled by microwave-assisted hydrodistillation (MWHD).

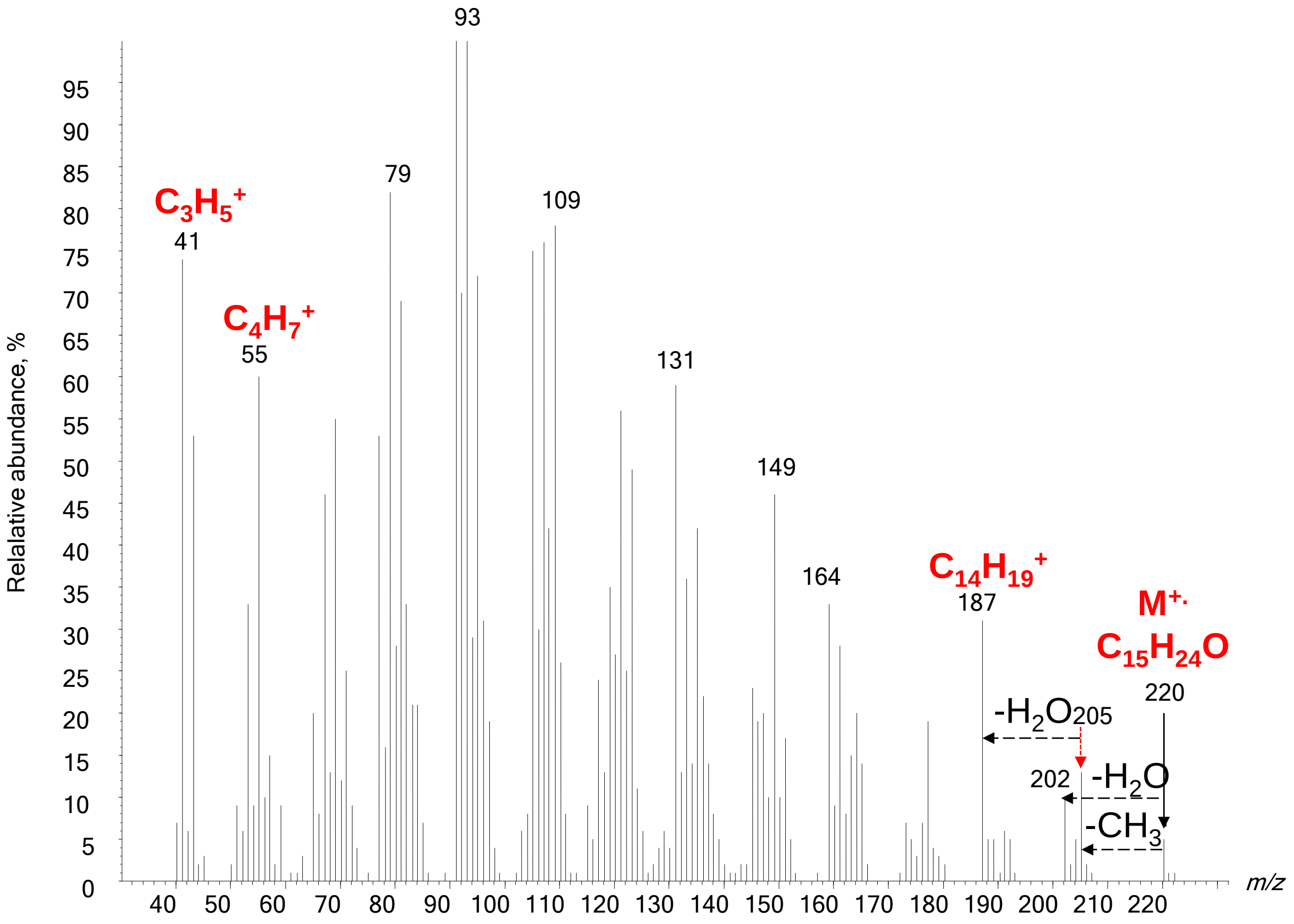

Figure S2. Mass spectrum obtained by GC/MS (EI, 70 eV) a sesquiterpenoid (peak N° 24 in **Table 2**, LRI 1683) in the *S. aratocensis* EO distilled by microwave-assisted hydrodistillation (MWHD).

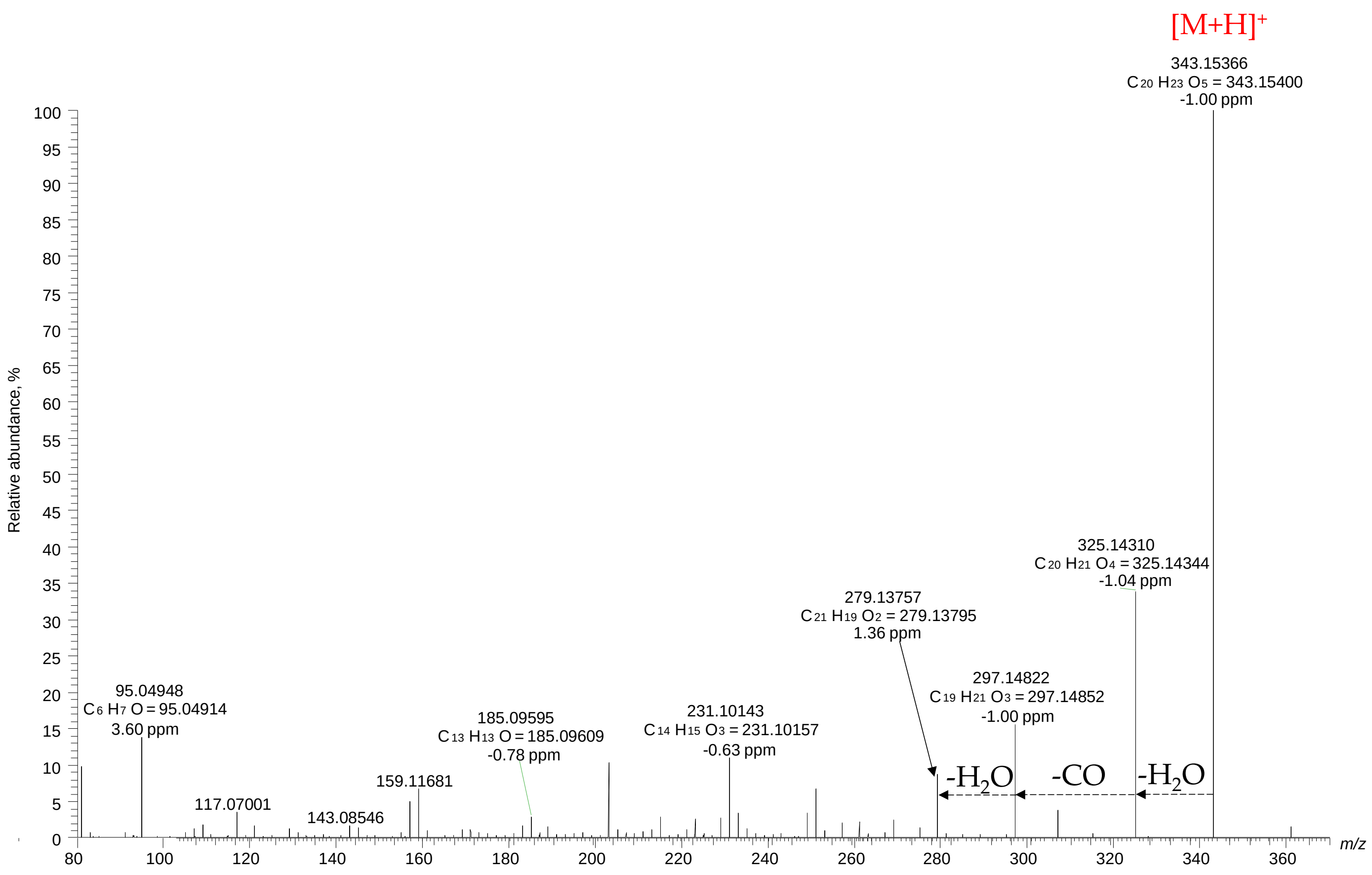

Figure S3. Mass spectrum obtained by UHPL-ESI(+)-*Orbitrap*-MS (SIM, *m/z* 343; HCD, 20 eV) of the compound N° 11 (**Table 3**) present in the hydroethanolic extract of *S. aratocensis* obtained from fresh plant before distillation.

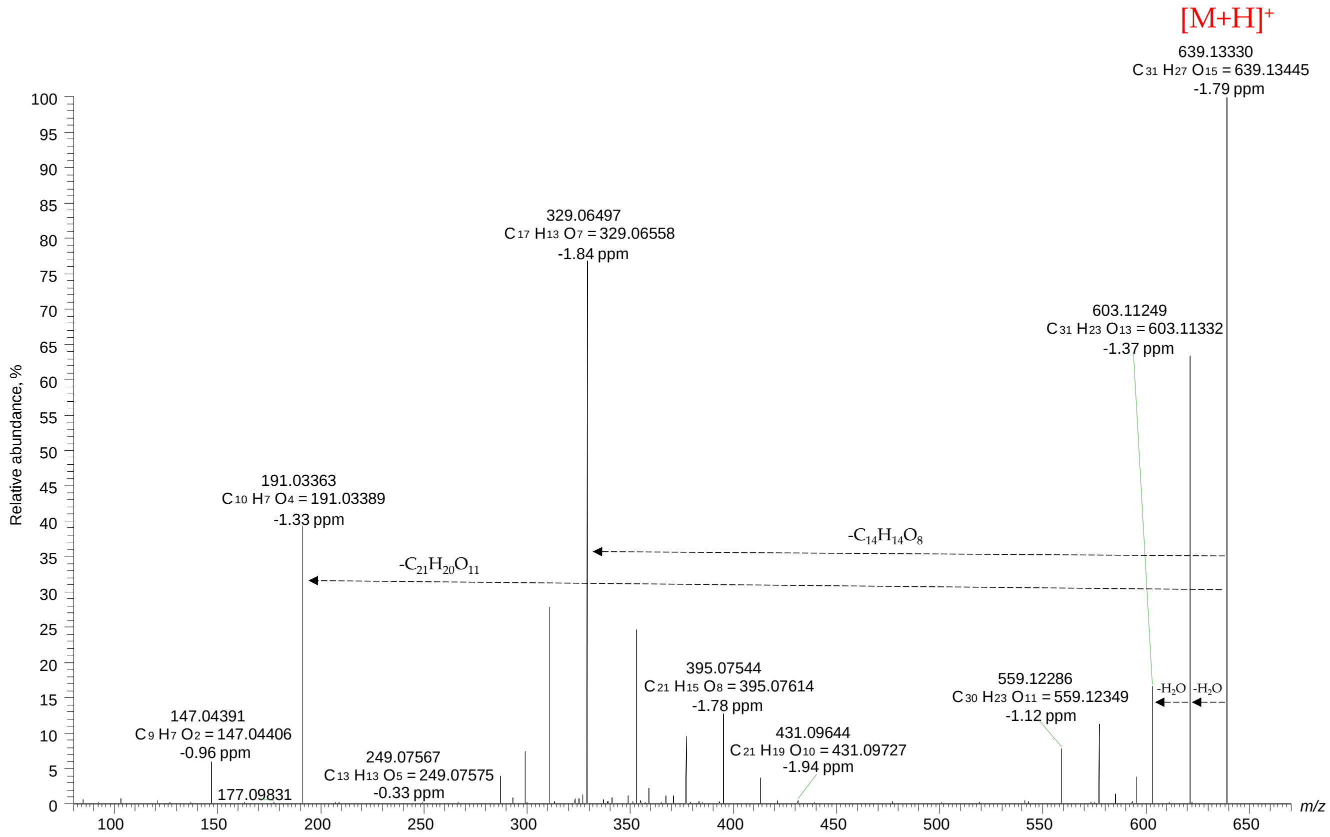

Figure S4. Mass spectrum obtained by UHPL-ESI(+)-*Orbitrap*-MS (SIM, *m/z* 639; HCD, 20 eV) of the compound N° 15 (**Table 3**) present in the hydroethanolic extract of *S. aratocensis* obtained from fresh plant before distillation.

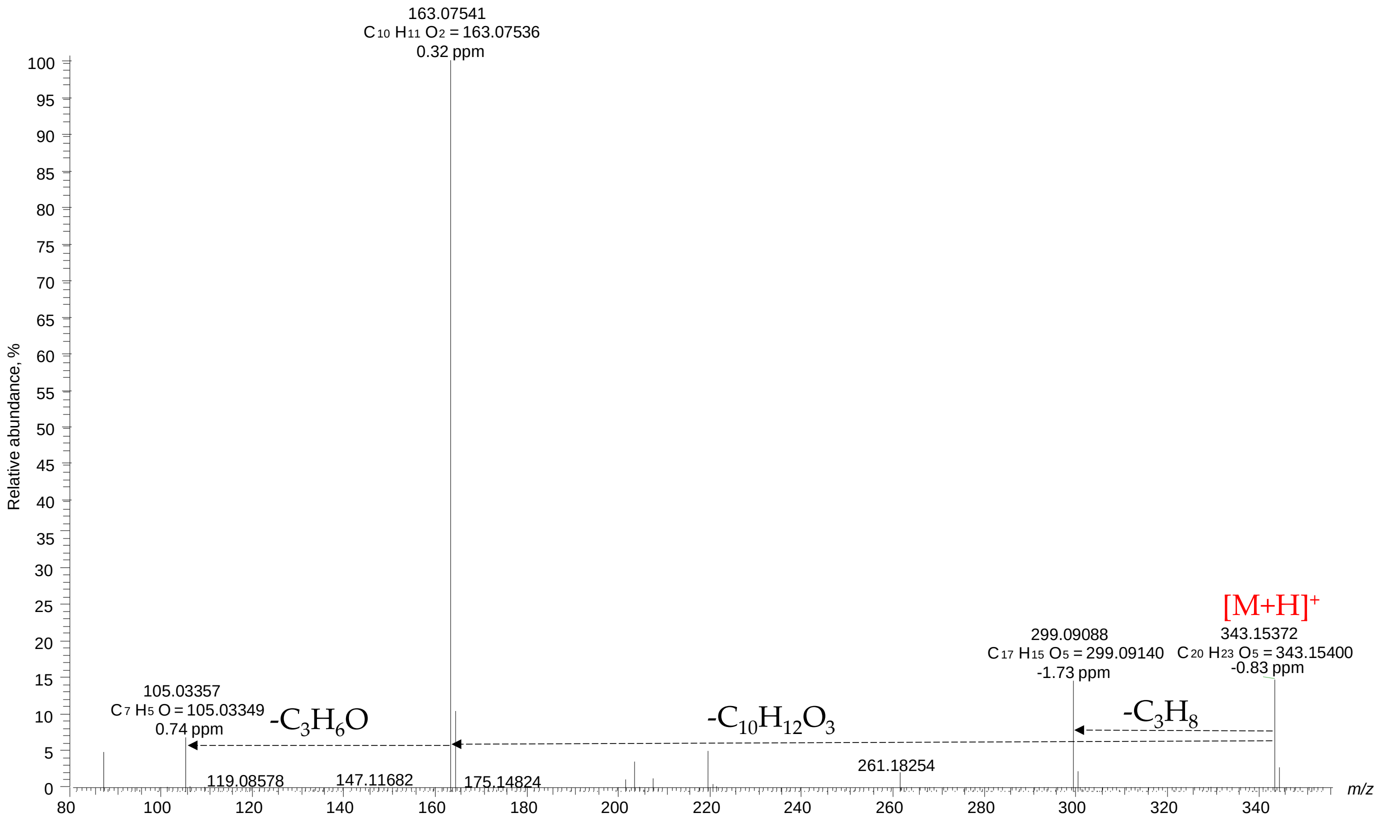

Figure S5. Mass spectrum obtained by UHPL-ESI(+)-*Orbitrap*-MS (SIM, *m*/*z* 343; HCD, 20 eV) of compound N° 23 (**Table 3**) present in the hydroethanolic extract of *S. aratocensis* obtained from vegetal material before distillation.
